# W. H. Elliot's Improved Method of Pivoting Teeth

**Published:** 1842-09

**Authors:** 


*W. H. Elliot's Improved Method of Pivoting Teeth.*—In a former number of our Journal we took occasion to notice the methods proposed by Dr. L. S. Parmly and Mr. S. L. Stringfellow, for giving egress to accumulations of purulent matter in the canal of the root of a tooth on which an artificial crown is engrafted. We stated that that of the former consisted in cutting a groove on the side of the pivot, and that that of the latter consisted in the formation of one on the side of the canal, occupied by the pivot in the root. The plan proposed by Dr. Elliot consists in the passing of a small gold tube through the centre of the pivot and in having an opening continuous with it, through the crown of the artificial tooth. We think this preferable to either of the other methods, and would recommend it to the attention of the profession.

Accompanying the crown of a porcelain tooth, pivoted in the manner just described, which Mr. E. had the kindness to present to us through the courtesy of Dr. McPhail, of the United States Army, was the crown of a central incisor, with a filling in it, and through the centre of which, is a gold tube, leading to the pulp-cavity of the tooth. The object of this is to give vent to any matters that may form in the cavity or canal of the tooth. This practice we believe originated with Dr. L. S. Parmly, or, at any rate, he described it to us twelve years ago, and we have had occasion to adopt it in many instances. It is however, only in the fewest number of cases that it should be had recourse to. H.

